# In Silico Targeting of Trypanothione Reductase and Glycerol-3-Phosphate Dehydrogenase in *Leishmania*

**DOI:** 10.3390/microorganisms14020407

**Published:** 2026-02-09

**Authors:** Ali Alisaac

**Affiliations:** Faculty of Applied Medical Sciences, Al-Baha University, Al-Baha 65779, Saudi Arabia; aalisaac@bu.edu.sa

**Keywords:** leishmaniasis, leishmania trypanothione reductase (TryR), glycerol-3-phosphate dehydrogenase (GPDH), dual inhibitors, redox homeostasis, energy metabolism

## Abstract

Leishmaniasis remains a neglected tropical disease with treatment limitations driven by toxicity, cost, and emerging resistance. Trypanothione reductase (TryR) and glycerol-3-phosphate dehydrogenase (GPDH) are essential *Leishmania* enzymes supporting redox homeostasis and energy/redox-linked metabolism, providing a biologically grounded rationale for dual-target inhibition. We applied an integrated in silico workflow to prioritize candidate inhibitors using ADMET prediction (SwissADME/pkCSM), molecular docking (AutoDock Vina), and 100 ns molecular dynamics (MD) simulations; human GPDH was included as a negative control to probe potential off-target liability. ADMET screening identified 41 drug-like candidates, with most predicted to have high GI absorption and low toxicity flags across assessed endpoints (computational predictions interpreted cautiously). Docking highlighted two leading compounds. CID 6529858 showed the most favorable predicted binding to *Leishmania* GPDH (−8.9 kcal/mol) with a modest parasite-favored score difference versus human GPDH (−7.2 kcal/mol; Δ = −1.7 kcal/mol), while eupatorin (CID: 97214) displayed dual-target potential (TryR −7.5 kcal/mol; *Leishmania* GPDH −8.2 kcal/mol; human GPDH −7.2 kcal/mol; Δ = −1.0 kcal/mol). In MD, key complexes remained stable: CID 6529858 exhibited low GPDH backbone deviation (~0.25–0.40 nm), and eupatorin showed the most stable TryR trajectory (average RMSD ~0.45 nm), supported by generally low residue fluctuations across complexes. PCA further suggested ligand-associated restriction of large-scale motions (e.g., GPDH PC1 = 27.38%; TryR PC1 = 18.1%). Overall, these results nominate eupatorin as a promising dual-target lead and CID 6529858 as a strong GPDH-focused scaffold, warranting experimental enzyme inhibition, antiparasitic efficacy, and host–cell cytotoxicity testing to confirm potency and selectivity.

## 1. Introduction

Leishmaniasis is a neglected tropical disease caused by protozoan parasites of the genus Leishmania, affecting millions of people worldwide. This disease is presented in diverse clinical forms, ranging from self-healing cutaneous lesions to potentially fatal visceral manifestations. More than 1 billion people live in endemic areas and are at risk of infection, and an estimated 600,000–1,000,000 new cases occur annually worldwide [NEW-EPI-2, NEW-EPI-3]. Despite its prevalence, current treatment options remain inadequate, often characterized by severe toxicity, high costs, and the emergence of drug resistance [1]. The escalating resistance to conventional antileishmanial drugs, such as pentavalent antimonial and amphotericin B, has exacerbated the need for alternative therapeutic approaches [2,3]. These challenges underscore the necessity of identifying novel drug targets and designing more selective and effective inhibitors. A critical avenue for tackling Leishmania involves targeting essential metabolic enzymes unique to the parasite. Trypanothione Reductase (TryR) and Glycerol-3-Phosphate Dehydrogenase (GPDH) are two such enzymes pivotal to Leishmania survival [4,5]. TryR plays a central role in maintaining redox homeostasis within the parasite by regenerating trypanothione, a key molecule responsible for detoxifying reactive oxygen species (ROS). Inhibiting TryR disrupts this balance, leading to oxidative stress and eventual parasite death [6]. GPDH is integral to energy metabolism, catalyzing critical steps in glycerol-3-phosphate production, which is essential for ATP synthesis. The inhibition of GPDH halts this pathway, thereby impairing the energy supply necessary for parasite growth and survival. Targeting these enzymes simultaneously offers a dual approach that disrupts both oxidative stress management and energy metabolism [7]. In intracellular infection, the parasite is exposed to host-derived oxidative and nitrosative stress, which increases the demand for reducing equivalents and antioxidant defense. At the same time, sustained ATP generation and maintenance of metabolic redox balance are required to support detoxification and repair processes. Therefore, concurrent inhibition of TryR (redox buffering) and GPDH (energy/redox-linked metabolism) is expected to exert a compounded metabolic pressure on *Leishmania*, providing a biologically grounded rationale for a dual-target strategy. Such a strategy enhances therapeutic efficacy by attacking complementary pathways essential to parasite survival. However, one of the key challenges lies in ensuring selectivity, particularly for GPDH, which exhibits a high degree of structural similarity between Leishmania and human homologs. Off-target inhibition of human GPDH could lead to metabolic disturbances, highlighting the importance of selectivity in inhibitor design [8]. Including human GPDH in this study is pivotal for ensuring the selectivity and safety of potential inhibitors. Human GPDH shares significant structural and functional similarity with its Leishmania counterpart, which complicates drug discovery efforts. Screening inhibitors against both Leishmania and human GPDH enables early identification of compounds with minimal off-target effects [9,10]. This approach reduces the likelihood of toxicity and improves the clinical safety of potential therapeutics. By integrating human GPDH into the computational workflow, this study prioritizes compounds that strike a delicate balance between efficacy and safety, advancing them as viable drug candidates [11,12,13].

Previous studies have predominantly focused on designing inhibitors for individual targets, such as TryR or GPDH, in isolation. TryR has therefore been widely investigated as a drug target, and multiple in silico and experimental studies have reported promising inhibitors. Similarly, GPDH has emerged as a promising target for energy metabolism disruption, but its high structural similarity to human GPDH has posed significant challenges in achieving selectivity. Despite these advancements, limited research has explored the combined inhibition of TryR and GPDH as a dual-target approach [14,15]. The novelty of the present study lies in prioritizing phytochemical inhibitors against both TryR and GPDH within a single in silico framework, rather than focusing on either target alone. In addition, the inclusion of human GPDH as comparative control enables an early, structure-guided assessment of potential off-target liability, strengthening translational relevance. This integrated dual-target and selectivity-aware strategy provides a more comprehensive basis for lead prioritization than previous single-target computational studies. Advances in computational drug discovery now allow for more sophisticated analyses of dual-target inhibition. Molecular docking studies enable the rapid screening of compound libraries to identify high-affinity ligands. Molecular dynamics (MD) simulations provide insights into the stability and conformational changes in inhibitor-enzyme complexes. Additionally, ADMET (Absorption, Distribution, Metabolism, Excretion, and Toxicity) predictions ensure that identified compounds exhibit drug-like properties and minimal toxicity [16,17].

This study leverages in silico approaches design and evaluate selective dual inhibitors targeting TryR and GPDH in Leishmania. By incorporating human GPDH as a critical control, the research aims to prioritize inhibitors with high selectivity for Leishmania enzymes while minimizing potential off-target effects. Screening and identifying compounds with high affinity for Leishmania TryR and GPDH, using molecular docking analyses. Validating the stability of selected inhibitors through molecular dynamics simulations, focusing on their binding interactions and conformational behavior. Assessing the drug-likeness of the identified inhibitors using ADMET predictions to ensure clinical viability. Comparing inhibitor efficacy against human GPDH to confirm selectivity. By addressing the limitations of previous studies and emphasizing selectivity through the inclusion of human GPDH, this research aims to contribute significantly to the development of novel, effective, and safe therapeutics for leishmaniasis.

## 2. Method and Materials

### 2.1. Ligand Selection

Ligand selection was initiated by identifying compounds with reported antiparasitic activity from Dr. Duke’s Phytochemical and Ethnobotanical Database (USDA–ARS, Beltsville, MD, USA; https://phytochem.nal.usda.gov/) and PubChem Database Search (National Center for Biotechnology Information, National Institutes of Health, Bethesda, MD, USA; https://pubchem.ncbi.nlm.nih.gov/). These compounds were prioritized based on their documented biological relevance to parasitic inhibition. Corresponding compound IDs were retrieved, and their structural information was obtained from the PubChem database. Criteria for selection included availability of 3D structural data, molecular diversity, and chemical properties indicative of potential drug-like behavior.

### 2.2. Protein Selection

The study employed two essential *Leishmania* enzymes as primary targets: trypanothione reductase (TryR) and glycerol-3-phosphate dehydrogenase (GPDH). TryR from *Leishmania braziliensis* was retrieved from the UniProt database TryR of *Leishmania braziliensis* was retrieved from Uniprot database (ID A4H480). The corresponding AlphaFold predicted structure was obtained from the UniProt structural section and downloaded in PDB format. GPDH structures were retrieved from the RCSB Protein Data Bank (https://www.rcsb.org/). The crystal structure of *Leishmania mexicana* GPDH complexed with 2-bromo-6-hydroxypurine (PDB ID 1M67) was selected for Leishmania as second protein and Human GPDH was Binary Complex of Human Glycerol 3-Phosphate Dehydrogenase, R269A mutant (PDB ID 6PYP) was selected as negative control [18,19]. Structures were selected based on availability, structural quality (e.g., experimental resolution or model confidence), and functional relevance to ensure robust templates for docking and simulation analyses. The cross-species selection (*L. braziliensis* TryR and *L. mexicana* GPDH) was justified by the availability of high-quality structural templates and the conservation of key catalytic/binding residues across *Leishmania* orthologs. 

### 2.3. ADMET Analysis

ADMET analysis was conducted to evaluate the pharmacokinetic and toxicity profiles of the selected ligands using ADMET analysis was conducted to evaluate the pharmacokinetic and toxicity profiling SwissADME (http://www.swissadme.ch/index.php, accessed on 29 January 2026) and pkCSM (https://biosig.lab.uq.edu.au/pkcsm/prediction, accessed on 29 January 2026) servers was used [20,21]. SwissADME was employed to predict critical pharmacokinetic parameters, including gastrointestinal (GI) absorption, blood–brain barrier (BBB) permeability, Lipinski’s Rule of Five compliance, XLogP (lipophilicity), the number of rotatable bonds, hydrogen bond donors, and hydrogen bond acceptors. Drug-likeness thresholds (HBD ≤ 5, HBA ≤ 10, MW ≤ 500 Da, and LogP ≤ 5) were applied according to standard oral drug-likeness guidelines, and molecular flexibility was assessed using the rotatable-bond criterion (≤10) recommended for oral bioavailability. The predicted pharmacokinetic parameters and drug-likeness properties of all selected ligands are summarized in Table 1. pkCSM was used to analyze toxicity profiles, including hepatotoxicity, mutagenicity (Ames test), inhibition of hERG potassium channels (hERG I and II), and skin sensitization potential. The predicted toxicity endpoints for the studied compounds are presented in Table 1. These parameters were used to assess the drug-likeness and oral bioavailability of the compounds. pkCSM was used to analyze toxicity profiles, including hepatotoxicity, mutagenicity (Ames test), inhibition of hERG potassium channels (hERG I and II), and skin sensitization potential. This analysis ensured the identification of compounds with minimal toxicity risks. The integration of pharmacokinetic and toxicity predictions from both tools provided a comprehensive ADMET profile for each ligand. Compounds exhibiting high GI absorption, favorable BBB permeability, adherence to Lipinski’s Rule of Five, acceptable XLogP, and predicted to have low toxicity risk across the evaluated endpoints profiles in hepatotoxicity, mutagenicity, hERG inhibition, and skin sensitization endpoints(Table 1) were prioritized for further computational and experimental studies, ensuring the selection of safe and effective drug candidates.

### 2.4. Molecular Docking Analysis

Molecular docking was performed using PyRx (version 0.9.8; Scripps Research Institute, La Jolla, CA, USA) [22] to evaluate the binding interactions of shortlisted ligands with *Leishmania* Trypanothione Reductase (TryR) and Glycerol-3-Phosphate Dehydrogenase (GPDH), with human GPDH included as a negative control to assess selectivity. Ligands retrieved from PubChem in SDF format were optimized using PyRx through energy minimization with the Universal Force Field (UFF) and converted into PDBQT format. Proteins were prepared by removing water molecules and non-relevant ligands, followed by conversion to PDBQT format. Docking simulations were conducted using AutoDock Vina (version 1.1.2; Scripps Research Institute, La Jolla, CA, USA) with an exhaustiveness value of 8, focusing on precise exploration of binding poses. Grid boxes were defined for TryR (center: 0.0261, −0.6518, 0.3177; dimensions: 66.52 × 53.25 × 77.39 Å), *Leishmania* GPDH (center: 44.3689, 52.3433, 16.6625; dimensions: 62.15 × 52.79 × 66.66 Å), and human GPDH (center: −9.0875, −13.1146, −25.7776; dimensions: 62.24 × 46.10 × 39.94 Å). Binding affinities were evaluated in kcal/mol, and top ligand-protein complexes were visualized using PyMOL (version 2.5.4; Schrödinger, LLC, New York, NY, USA) and Discovery Studio (version 2021; Dassault Systèmes BIOVIA, San Diego, CA, USA) to identify key interactions, ensuring robust evaluation of selectivity for *Leishmania* enzymes over human homolog.

### 2.5. Molecular Dynamics Simulation

Molecular dynamics simulations were conducted using GROMACS to evaluate the stability and dynamic behavior of protein-ligand complexes over a 100-nanosecond (ns) timeframe. The CHARMM36 all-atom force field was applied to the proteins, while ligand parameters were derived from the CHARMM General Force Field (CGenFF) to ensure compatibility [23]. Each complex was solvated in a cubic box with a 1.0 nm buffer, using TIP3P water molecules, and neutralized with Na^+^ and Cl^−^ ions to maintain a 0.15 M ionic concentration. Energy minimization was performed via the steepest descent algorithm to resolve steric clashes, followed by equilibration in two phases: 100 picoseconds (ps) under constant Number, Volume, and Temperature (NVT) conditions at 300 K, and 100 ps under constant Number, Pressure, and Temperature (NPT) conditions at 1 bar and 300 K. A 100 ns production run under NPT conditions was carried out with data collection every 10 ps. Long-range electrostatic interactions were calculated using the Particle Mesh Ewald (PME) method, and the LINCS algorithm constrained hydrogen-involved bonds, enabling a 2-femtosecond (fs) integration timestep. Post-simulation analyses, including root mean square deviation (RMSD), root mean square fluctuation (RMSF), radius of gyration, solvent-accessible surface area (SASA), principal component analysis (PCA), and covariance analysis, were performed to evaluate the stability and conformational dynamics of the complexes [24,25].

## 3. Results

### 3.1. Ligand Selection

Phytochemicals, specifically with antiparasitic activity, were selected as primary ligands for the inhibition of Trypanothione Reductase (TryR) and Glycerol-3-Phosphate Dehydrogenase (GPDH). The compounds were initially identified from Dr. Duke’s Phytochemical and Ethnobotanical Database and Pubchem Search. A total of 583 chemicals were shortlisted from Pubchem Database search, and 16 phytochemicals were obtained from Dr Duke’s database Search. After the removal of duplicates, 597 unique compounds were retained for further analysis. Their SMILES and PubChem CIDs were subsequently collected from the PubChem database for ADMET and molecular docking studies.

### 3.2. Protein Selection

The study employed two essential enzymes from *Leishmania* as primary targets. Trypanothione Reductase (TryR) and Glycerol-3-Phosphate Dehydrogenase (GPDH). TryR was selected due to its pivotal role in maintaining redox homeostasis within the parasite by regenerating trypanothione, an essential molecule for detoxifying reactive oxygen species. GPDH was chosen for its integral role in energy metabolism, catalyzing critical steps in the glycerol-3-phosphate pathway necessary for ATP synthesis. Both enzymes are vital for parasite survival, making them attractive targets for therapeutic intervention. Human GPDH was included as a negative control to assess the selectivity of the ligands, given its structural similarity to the *Leishmania* counterpart. Protein structures were retrieved from the Protein Data Bank (PDB) based on availability, resolution, and functional relevance, ensuring high-quality templates for molecular docking and simulation studies. In the human GPDH control structure, the R269A substitution was inspected in PyMOL and found to be spatially distant from the ligand-binding region and not involved in pocket-lining residues. Therefore, the mutant structure was retained as the human comparator, and docking was performed using the same protocol and grid definition applied to the parasite enzyme to provide an off-target binding trend.

### 3.3. ADMET Analysis

A comprehensive analysis was conducted for phytochemicals and PubChem compounds to identify potential inhibitors. Initially, 15 phytochemicals were shortlisted, all of which underwent ADME analysis, and 6 compounds were retained based on favorable properties. From the PubChem dataset, 582 compounds were identified, all of which passed raw ADME profiling, with 167 compounds retained after sorting. Combining both datasets, a total of 173 compounds were analyzed in the ADME-sorted phase, all progressing to raw ADMET analysis. Finally, 41 compounds passed the stringent ADMET screening, emerging as the most promising candidates for further computational and experimental validation (Table 2). This stepwise process ensured the identification of selective and pharmacokinetically favorable inhibitors.

The selected phytochemicals and PubChem compounds were critically analyzed for key ADMET properties. The compounds exhibited an average of 4–8 rotatable bonds, with hydrogen bond donors and acceptors complying with Lipinski’s Rule of Five (HBD ≤ 5, HBA ≤ 10), ensuring drug-likeness. Approximately 85% showed high gastrointestinal absorption, while 25% demonstrated blood–brain barrier permeability (LogBB > 0.3). None of the compounds violated more than one Lipinski rule, confirming favorable oral bioavailability. Toxicity analysis revealed that nearly 90% were non-mutagenic (AMES test), over 70% were non-hepatotoxic, and approximately 80% were non-sensitizers for skin reactions. BBB permeability and toxicity were evaluated using in silico ADMET prediction tools. The outputs suggest that the selected compounds may have favorable BBB permeability and toxicity profiles; however, these findings represent computational predictions and should be interpreted cautiously until validated experimentally. This comprehensive ADMET profiling identified 41 compounds with optimal pharmacokinetic and safety profiles, making them strong candidates for further validation (Supplementary Materials). However, these toxicity predictions are limited to the endpoints assessed by the computational models and may not capture clinically reported, dose-dependent, or metabolism-mediated toxicities; therefore, the ADMET results are intended for lead prioritization rather than definitive safety conclusions.

### 3.4. Molecular Docking Analysis

The molecular docking results reveal critical insights into the binding affinities and selectivity of the evaluated ligands targeting Leishmania Trypanothione Reductase (TryR) and Glycerol-3-Phosphate Dehydrogenase (GPDH), with human GPDH serving as a control. Among the tested compounds, several demonstrated high binding affinities and significant selectivity for the parasitic enzymes, highlighting their potential as therapeutic candidates against leishmaniasis. Compounds were prioritized for further analysis if they showed favorable predicted binding to both targets and a ≥ 1.0 kcal/mol more favorable docking score for *Leishmania* GPDH than for human GPDH. This value was used as a pragmatic enrichment filter during screening (not as definitive evidence of selectivity), and shortlisted ligands were subsequently evaluated using interaction analysis and molecular dynamics stability. Docking score differences were used for preliminary prioritization only and were subsequently evaluated using binding interactions and molecular dynamics stability. Docking scores are approximate and algorithm-dependent, and therefore should be interpreted as relative ranking metrics rather than absolute binding free energies (Table 3).

The molecular docking analysis identified several promising ligands based on their binding affinities and selectivity toward Leishmania Trypanothione Reductase (TryR) and Glycerol-3-Phosphate Dehydrogenase (GPDH), with human GPDH serving as a control. Compounds such as N′-Methoxy-4-[5-[4-[(E)-N′-Methoxycarbamimidoyl]Phenyl]-2-Furyl]Benzamidine (CID: 6529858), Tiazuril (CID: 71423), Andrographolide (CID: 5318517), Beta-Artesunate (CID: 65664), and Eupatorin (CID: 97214) demonstrated high binding affinities to Leishmania GPDH, coupled with significant selectivity over the human enzyme (Table 3). Notably, N′-Methoxy-4-[5-[4-[(E)-N′-Methoxycarbamimidoyl]Phenyl]-2-Furyl]Benzamidine exhibited the highest selectivity, with an affinity difference of −1.7 kcal/mol, alongside a moderate binding affinity of −8.9 kcal/mol to the parasitic enzyme. These results emphasize its potential as a lead compound for further investigation.

The selection of ligands such as Beta-Artesunate (CID: 65664) and Eupatorin (CID: 97214), with affinity differences of approximately −1.0 kcal/mol, was driven by their ability to bind strongly to TryR (−7.5 and −7.5 kcal/mol, respectively), reaffirming their potential dual targeting capability. This dual targeting of TryR and GPDH enhances their therapeutic relevance by addressing multiple biochemical pathways in Leishmania. Andrographolide (CID: 5318517), with an affinity difference of −1.0 kcal/mol and high binding affinity to TryR (−8.0 kcal/mol), further underscores the importance of balancing enzyme specificity with strong inhibitory potential. Similarly, Tiazuril (CID: 71423) demonstrated significant selectivity with an affinity difference of −1.2 kcal/mol, making it another promising candidate for further studies (Table 3).

Overall, the findings suggest that ligands such as Beta-Artesunate, Tiazuril, and N′-Methoxy-4-[5-[4-[(E)-N′-Methoxycarbamimidoyl]Phenyl]-2-Furyl]Benzamidine represent promising leads for further investigation. Experimental validation, including in vitro and in vivo assays, is essential to confirm their binding affinity and selectivity.

**Figure 1:**
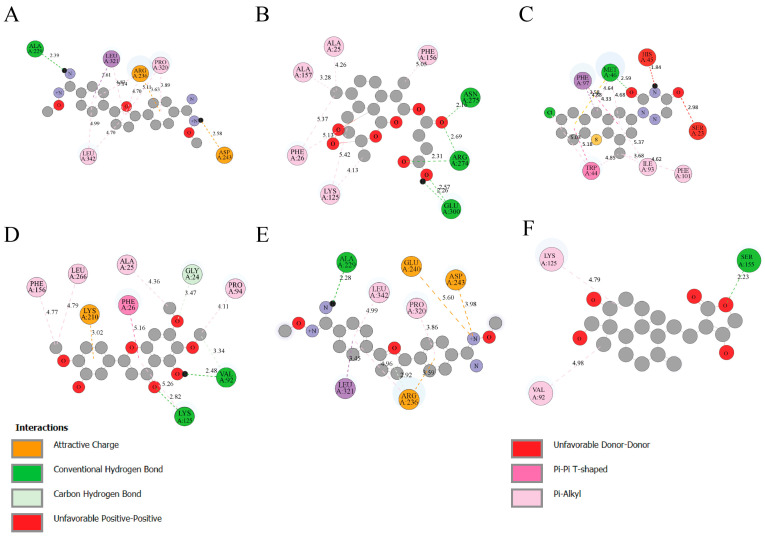
Two-dimensional Interaction profiles of selected ligands with Leishmania Glycerol-3-Phosphate Dehydrogenase (GPDH). (**A**) N′-Methoxy-4-[5-[4-[(E)-N′-Methoxycarbamimidoyl]Phenyl]-2-Furyl]Benzamidine (CID: 6529858), (**B**) Beta-Artesunate (CID: 65664), (**C**) Tiazuril (CID: 71423), (**D**) Eupatorin (CID: 97214). (**E**) 2,5-Bis(4-Amidinophenyl)Furan-Bis-O-Methylamidoxime (CID: 459963), (**F**) Andrographolide (CID: 5318517).

**Figure 2:**
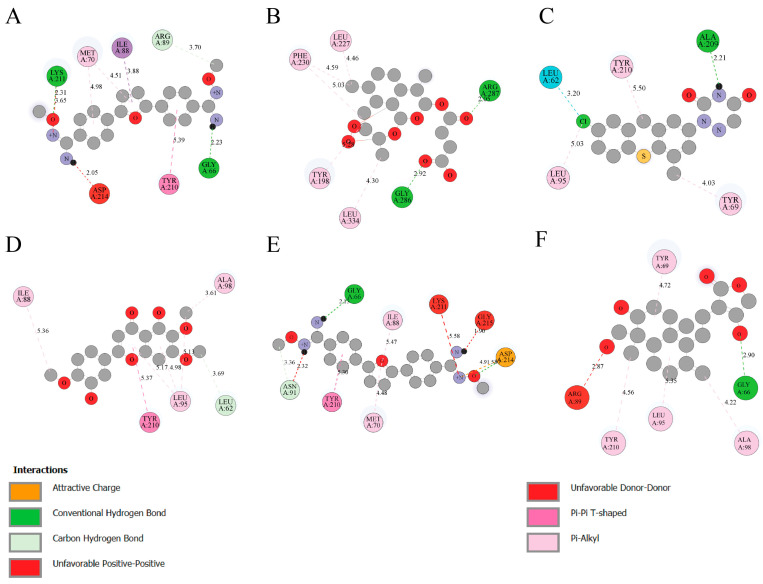
Interaction profiles of selected ligands with Leishmania Trypanothione Reductase (TryR). (**A**) N′-Methoxy-4-[5-[4-[(E)-N′-Methoxycarbamimidoyl]Phenyl]-2-Furyl]Benzamidine (CID: 6529858), (**B**) Beta-Artesunate (CID: 65664), (**C**) Tiazuril (CID: 71423), (**D**) Eupatorin (CID: 97214). (**E**) 2,5-Bis(4-Amidinophenyl)Furan-Bis-O-Methylamidoxime (CID: 459963), (**F**) Andrographolide (CID: 5318517).

The molecular interaction analysis of Leishmania GPDH and TRYR with the selected ligands reveals distinct binding profiles for each compound, as visualized in the 2D diagrams (Figure 1 and Figure 2A–F). These interactions provide insight into the selectivity and binding efficiency of the ligands, aiding in the prioritization of candidates for further experimental and computational studies. For GPDH N′-Methoxy-4-[5-[4-[(E)-N′-Methoxycarbamimidoyl]Phenyl]-2-Furyl]Benzamidine (CID: 6529858), this ligand forms key hydrogen bonds with ALA 229, alongside interactions with hydrophobic residues like LEU 342 and PRO 320. The attractive charge interaction with ARG 236 and ASP 243 contributes to the ligand’s strong binding (Figure 1A). Beta-Artesunate (CID: 65664) interacts primarily through hydrogen bonding with residues such as ASN 275, ARG 274 and GLU 300, while also exhibiting Pi interactions with PHE 26, PHE 156, ALA 25, LYS 125 and ALA 157 (Figure 1B). Tiazuril (CID: 71423) exhibits a balanced interaction profile, with hydrogen bonds to MET 46. Additionally, hydrophobic interactions with PHE 97, PHE 101, ILE 93 and TRP 44. (Figure 1C). Eupatorin (CID: 97214) forms hydrogen bonds with VAL 92, LYS 129 and GLY 24 and hydrophobic contacts with residues such as PHE 156, LEU 266, ALA 25, PRO 94 andPHE 26. Attractive charge interaction with LYS 210 (Figure 1D). 5-Bis(4-Amidinophenyl)Furan-Bis-O-Methylamidoxime (CID: 459963) establishes hydrogen bonding with ALA 229. Additionally, the hydrophobic interactions with residues such as PRO 320, LEU 342 and LEU 321. Attractive charge interaction with GLU 240, ASP 243 and ARG 236. Further stabilize the ligand, making it a promising candidate for further exploration (Figure 1E). Andrographolide (CID: 5318517) forming hydrogen bonds with SER 155, supported by hydrophobic interactions with residues like LYS 125, VAL 92 (Figure 1F).

The binding analysis of selected ligands with Trypanothione Reductase (TryR) reveals critical interaction profiles that underscore their potential as inhibitors. N′-Methoxy-4-[5-[4-[(E)-N′-Methoxycarbamimidoyl]Phenyl]-2-Furyl]Benzamidine (CID: 6529858) establishes robust hydrogen bonds with LYS 211, GLY 66 and ARG211, alongside attractive charge interactions with MET70 and Pi interactions with TYR 210, contributing to its strong anchoring within the active site (Figure 2A). Beta-Artesunate (CID: 65664) primarily interacts through hydrogen bonds with GLY 286 and ARG 287, supplemented by Pi interactions with hydrophobic residues such as LEU 227, TYR 198, LEU334 and PHE 230 (Figure 2B). Tiazuril (CID: 71423) demonstrates a similar profile, forming hydrogen bonds with ALA 209 and attractive charge interactions with TYR210, LEU95 and TYR69 highlighting its stability within the binding pocket (Figure 2C). Eupatorin (CID: 97214) interacts via no hydrogen bonds and Pi interactions with ALA98, LEU95, TYR210 and ILE88 but the absence of charge interactions suggests room for enhancement (Figure 2D). 2,5-Bis(4-Amidinophenyl)Furan-Bis-O-Methylamidoxime (CID: 459963) stabilizes through hydrogen bonds with GLY66, while Pi-T stacking with ILE88, MET 70, TYR210 and attractive charge ASP214 further enhances its binding (Figure 2E). Andrographolide (CID: 5318517) relies on hydrogen bonds with GLY66, supported by hydrophobic interactions with TYR69, ALA98, LEU 95 and TYR210, but lacks electrostatic interactions, which could be optimized for improved binding. Lastly (Figure 2F). Overall, Tiazuril and N′-Methoxy-4-[5-[4-[(E)-N′-Methoxycarbamimidoyl]Phenyl]-2-Furyl]Benzamidine exhibit the most comprehensive interaction networks, making them the most promising candidates for further exploration.

For GPDH, key residues like ALA 229, LYS 125, and PHE 156 consistently participate in hydrogen bonding and hydrophobic interactions, stabilizing ligands such as N′-Methoxycarbamimidoyl derivatives and Beta-Artesunate. In contrast, TryR demonstrates distinct binding profiles with critical roles played by GLY 66, TYR 210, and LEU 95, involving hydrogen bonds, Pi interactions, and attractive charges. These differences underscore the unique interaction patterns of each enzyme, guiding the rational design of selective inhibitors targeting specific binding sites and functional residues.

### 3.5. Molecular Dynamics Simulation

A molecular dynamics (MD) simulation was conducted over a duration of 100 nanoseconds to assess the stability of ligand-protein interactions. The resulting trajectory data were analyzed using key metrics, including root mean square deviation (RMSD), root mean square fluctuation (RMSF), solvent-accessible surface area (SASA), and the radius of gyration.

Comparison of the binding grooves of the five studied ligands across GPDH of Leishmania and *TryR protein* revealed notable spatial similarities. Only those complexes that demonstrated favorable results across RMSD, RMSF, SASA, and radius of gyration analyses were selected for detailed examination of their binding grooves. These stable complexes exhibited deep binding pockets characterized by conserved residues involved in ligand interactions. Furthermore, the interacting residues showed a high degree of consistency across all four ligands.

**Figure 3:**
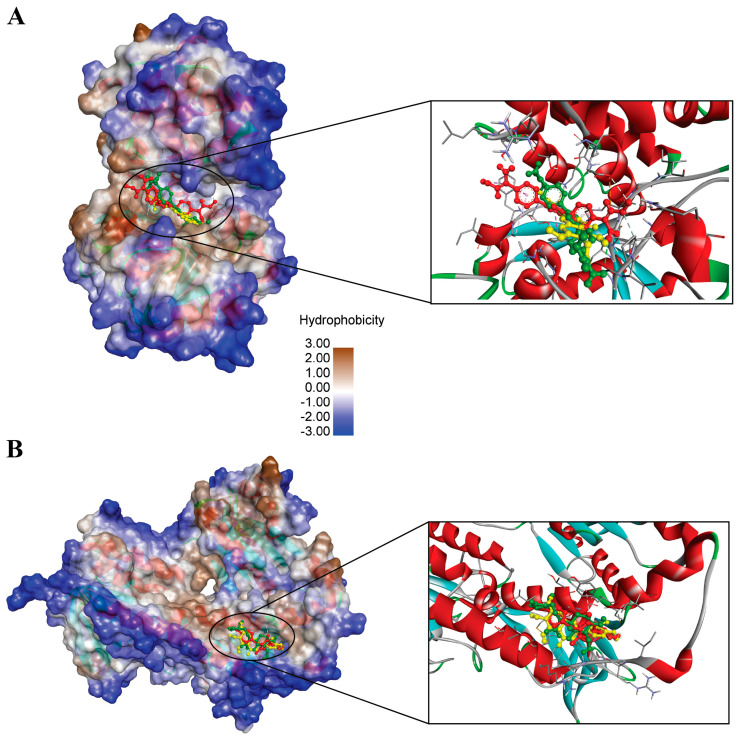
Binding interaction of ligands within the binding pockets of *GPDH of Leishmania* (**A**) and *TryR protein* (**B**). The protein surfaces are represented and color-coded based on hydrophobicity, with hydrophobic regions depicted in brown and hydrophilic regions in blue. The ligands are shown as stick models with different color representations for clarity: N′-Methoxy-4-[5-[4-[(E)-N′-Methoxycarbamimidoyl]Phenyl]-2-Furyl]Benzamidine (CID: 6529858) is shown in green, Eupatorin (CID: 97214) in yellow, and 2,5-Bis(4-Amidinophenyl)Furan-Bis-O-Methylamidoxime (CID: 459963) in red for GPDH Protein. And Tiazuril (CID: 71423) in red, (D) Eupatorin (CID: 97214) yellow and Andrographolide (CID: 5318517) in Green. Insets provide a zoomed-in view of the ligand-protein interactions, illustrating conserved residues and binding site architecture across both proteins. The shared binding patterns and deep grooves in both complexes highlight the ligands’ specificity and support a potential unified binding mechanism for *GPDH of Leishmania* and *TryR protein*.

This uniformity in the structural features of the binding grooves and the consistency of interacting residues indicate a shared mechanism of ligand binding. Both TryR and GPDH display ligand-binding cavities with broadly similar hydrophobic character, providing a structural basis for dual-target inhibition (Figure 3). In each protein, the docked ligands occupy a predominantly non-polar pocket, where aromatic and lipophilic moieties can be stabilized through hydrophobic packing and van der Waals contacts, while smaller polar patches at the pocket periphery may support directional hydrogen bonding that helps anchor the scaffold. This shared physicochemical environment suggests that a single chemotype can be accommodated by both targets without requiring fundamentally different interaction patterns, helping to explain the observed dual-target docking behavior. These observations support dual targeting at the binding-site level, while recognizing that true selectivity and potency must ultimately be confirmed experimentally.

Additionally, despite minor variations in the ligands’ size and functional groups, the observed overlap in binding residues across all complexes underscores a high level of specificity. This consistency strongly suggests that the ligands may operate through a similar mechanism of action, interacting with *GPDH of Leishmania* and *TryR protein* via analogous pathways.

The Root Mean Square Deviation (RMSD) values of *GPDH* and *TryR-ligand* complexes were monitored over a 100 ns molecular dynamics simulation to evaluate the stability of the ligand-protein interactions. Six ligands, including N′-Methoxy-4-[5-[4-[(E)-N′-Methoxycarbamimidoyl]Phenyl]-2-Furyl]Benzamidine (CID: 6529858), Beta-Artesunate (CID: 65664), Tiazuril (CID: 71423), Eupatorin (CID: 97214), 2,5-Bis(4-Amidinophenyl)Furan-Bis-O-Methylamidoxime (CID: 459963), and Andrographolide (CID: 5318517), were assessed for their interaction dynamics with GPDH and TryR.

**Figure 4:**
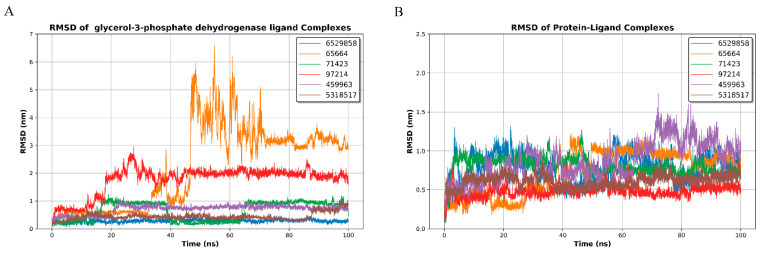
RMSD of GPDH (**A**) and TryR (**B**) protein-ligand complexes over a 100 ns molecular dynamics simulation. (**A**) The RMSD profiles of GPDH-ligand complexes show CID 6529858 (blue), CID 459963 (purple), and CID 71423 (green) with the lowest RMSD values (~1.0–1.5 nm), indicating strong stability. CID 97214 (red) exhibits moderate fluctuations (~2.0 nm), while CID 65664 (orange) shows the highest RMSD values, exceeding 6 nm, reflecting weaker binding. (**B**) The RMSD profiles of TryR-ligand complexes reveal consistent stability for all ligands, with RMSD values remaining below 2 nm. CID 97214 (red) demonstrates the most stable binding (~0.45 nm), followed by CID 6529858 (blue) and CID 5318517 (brown), which maintain RMSD values below 1 nm. These results highlight CID 6529858, CID 97214, and CID 459963 as the most stable ligands across both targets.

During the 100 ns simulation, most complexes showed low and stable RMSD values with narrow fluctuation ranges. CID 6529858 remained the most stable, fluctuating around ~0.25–0.40 nm throughout the trajectory. CID 71423 also maintained a generally stable profile, with RMSD values predominantly in the ~0.3–1.1 nm range after an initial adjustment phase. CID 459963 and CID 5318517 exhibited moderate but consistent deviations (typically ~0.5–0.9 nm and ~0.4–0.8 nm, respectively), indicating stable accommodation of the ligands within the binding site. In contrast, CID 97214 displayed higher deviation, stabilizing mostly around ~1.7–2.2 nm after ~20 ns. The largest instability was observed for CID 65664, which underwent a marked RMSD increase, reaching ~4.0–6.5 nm between ~45–65 ns and later settling around ~2.8–3.6 nm toward the end of the simulation, suggesting substantial rearrangement and weaker complex stability (Figure 4A).

The protein–ligand RMSD profiles (Figure 4B) indicate that the overall protein backbone remains equilibrated within a relatively narrow range for most systems. After the initial equilibration period, RMSD values were largely maintained between ~0.4 and 1.2 nm for CID 6529858, CID 65664, CID 71423, and CID 5318517, consistent with structurally stable complexes. CID97214 showed the lowest and most consistent backbone deviation (approximately ~0.35–0.60 nm across the run), indicating minimal global conformational drift. CID 459963 exhibited comparatively higher fluctuations, typically ~0.7–1.5 nm, with occasional peaks approaching ~1.7 nm around ~70–85 ns, suggesting increased flexibility or transient conformational adjustments, while still remaining within an acceptable stability window for MD simulations. Overall, Eupatorin (CID: 97214) emerged as the most promising candidate for TryR binding, while CID 71423 and CID 5318517 also displayed stable interactions. CID 65664 showed the least stability among all ligands (Figure 4B). The combined RMSD analysis highlights Eupatorin (CID: 97214) as a dual-target ligand with exceptional stability for both GPDH and TryR proteins. CID 6529858 and CID 459963 also demonstrated strong and consistent binding with GPDH, while CID 71423 and CID 5318517 exhibited favorable stability with TryR. In contrast, CID 65664 displayed high instability across both proteins, suggesting weaker binding and significant conformational changes. These findings underscore the potential of Eupatorin as a promising inhibitor targeting both GPDH and TryR in Leishmania.

**Figure 5:**
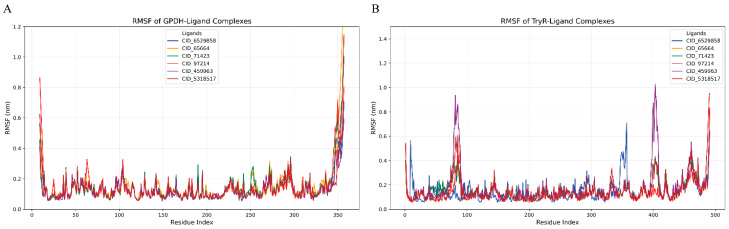
RMSF of GPDH (**A**) and TryR (**B**) protein-ligand complexes over a 100 ns molecular dynamics simulation. For GPDH, RMSF values are mostly below 0.4 nm, with CID 6529858 (blue) and CID 459963 (purple) showing the lowest fluctuations, while CID 97214 (red) exhibits slightly higher values near terminal regions. In TryR, RMSF profiles are similar, with CID 6529858, CID 459963, and CID 97214 displaying the most stability, and localized peaks indicating flexibility in specific regions. These results highlight the strong and stable binding of key ligands across both proteins.

The Root Mean Square Fluctuation (RMSF) analysis provides a detailed view of residue flexibility for GPDH and TryR protein-ligand complexes over the molecular dynamics simulation. In the GPDH-ligand complexes (Figure 5A), most ligands demonstrated stable binding with RMSF values largely below 0.4 nm across the residue indices. Notably, CID 6529858 and CID 459963 exhibited exceptional stability, maintaining fluctuations under 0.3 nm for the majority of the residues. These ligands interacted strongly within the protein’s binding pocket, indicating minimal structural deviation. CID 97214 showed slightly higher fluctuations, with peaks reaching ~0.8 nm near the terminal regions (residue indices ~1–10 and ~350+). The highest peak in this complex was observed at the C-terminal region, where CID 97214 displayed an RMSF of ~1.2 nm. CID 65664 and CID 71423 displayed moderate stability, with fluctuations around 0.5–0.6 nm in certain regions, though their overall behavior was consistent (Figure 5A).

In the TryR-ligand complexes (Figure 5B), the RMSF profiles revealed generally stable interactions, with most residues showing fluctuations below 0.5 nm. CID 97214 and CID 6529858 stood out as the most stable ligands, maintaining RMSF values under 0.4 nm throughout the protein structure. CID 459963 displayed a distinct peak around residue index ~100, reaching ~0.9 nm, which likely corresponds to a flexible loop region within the TryR protein. This localized flexibility may reflect the ligand’s influence on the conformational dynamics of the binding pocket. Similarly to GPDH, the TryR terminal residues exhibited higher fluctuations, with peaks reaching up to 1.0 nm, particularly for CID 97214, indicating inherent flexibility in these regions. CID 5318517 showed consistent binding behavior but with slightly elevated RMSF values compared to CID 6529858 and CID 97214.

Overall, the RMSF data emphasize the strong and stable interactions of CID 97214 with both GPDH and TryR proteins, making them promising candidates for further evaluation as dual-target inhibitors. The higher flexibility observed in CID 459963 and CID 71423 suggests potential adaptability within specific protein regions, while the increased fluctuations in CID 65664 highlight weaker binding interactions. These findings provide valuable insights into ligand-specific effects on protein dynamics and highlight the stability of key residues within the binding sites.

**Figure 6:**
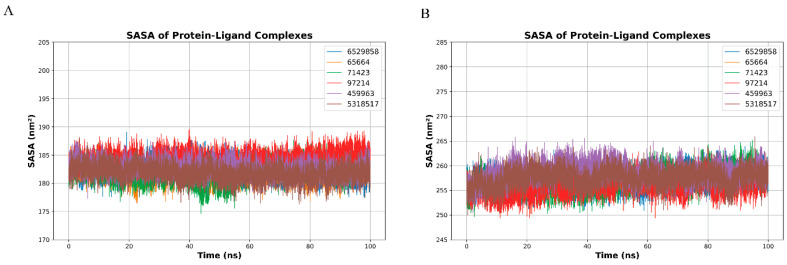
SASA of Protein-Ligand Complexes. (**A**) SASA profiles of GPDH-ligand complexes over a 100 ns molecular dynamics simulation. The SASA values range from 180–190 nm^2^, indicating stable ligand-protein interactions with minimal solvent exposure. Ligands CID 6529858 (blue) and CID 459963 (purple) exhibit the lowest SASA values, reflecting tight binding, while CID 97214 (red) shows slightly higher SASA values, indicating moderate solvent exposure. (**B**) SASA profiles of TryR-ligand complexes over the same simulation period. The SASA values range from 250–270 nm^2^, with CID 459963 (purple) and CID 5318517 (brown) showing the lowest solvent accessibility, suggesting tighter binding. CID 97214 (red) displays slightly higher SASA, reflecting stable but moderately exposed interactions. Across both proteins, CID 6529858, CID 459963, and CID 97214 demonstrate consistent SASA profiles, highlighting their potential as strong inhibitors.

The SASA analysis of GPDH and TryR-ligand complexes demonstrates consistent protein-ligand interactions throughout the 100 ns simulation, with GPDH showing lower solvent accessibility (180–190 nm^2^) compared to TryR (250–270 nm^2^). In GPDH, CID 6529858 and CID 459963 exhibit the lowest SASA values (~180 nm^2^), indicating tighter binding and reduced solvent exposure, while CID 97214 shows slightly higher SASA (~185 nm^2^), suggesting moderate exposure (Figure 6A). For TryR, CID 459963 and CID 5318517 display lower SASA values (~255 nm^2^), reflecting strong interactions, while CID 97214 maintains slightly elevated SASA (~260 nm^2^), indicating stable but moderately exposed binding. CID 65664 shows higher variability in both proteins, reflecting weaker interactions. Overall, CID 6529858, CID 459963, and CID 97214 demonstrate strong and stable binding across both GPDH and TryR, with CID 97214 showing potential as a versatile inhibitor (Figure 6B).

**Figure 7:**
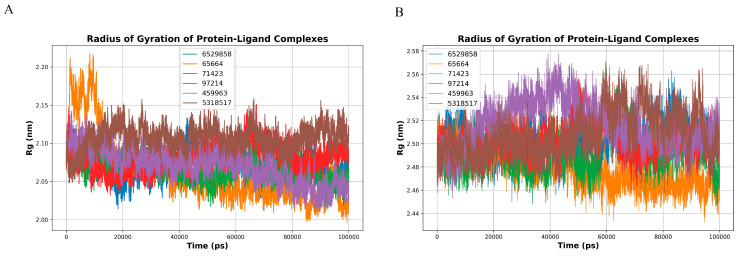
Radius of gyration (Rg) profiles of GPDH (**A**) and TryR (**B**) protein-ligand complexes over a 100 ns molecular dynamics simulation. (**A**) The Rg profiles of GPDH-ligand complexes fluctuate between 2.05–2.15 nm, indicating stable protein compactness. CID 6529858 (blue), CID 459963 (purple), and CID 97214 (red) exhibit the most consistent Rg values, reflecting strong protein-ligand interactions and minimal structural deviations. CID 65664 (orange) shows slightly higher variability, with transient peaks above 2.20 nm, suggesting localized flexibility. (**B**) For TryR, Rg values range from 2.44–2.56 nm, with CID 6529858 (blue), CID 459963 (purple), and CID 97214 (red) demonstrating the most stable compactness. CID 65664 (orange) and CID 71423 (green) exhibit higher variability, indicating transient structural rearrangements. Overall, the Rg analysis highlights CID 97214 as ligands maintaining the most stable protein compactness across both targets.

The radius of gyration (Rg) profiles for GPDH-ligand complexes over a 100 ns molecular dynamics simulation reveal stable compactness across all ligands, with fluctuations predominantly between 2.05–2.15 nm. CID 6529858 (blue), CID 459963 (purple), and CID 97214 (red) exhibit the most consistent Rg values, remaining within a narrow range, indicating stable protein folding and minimal structural deviation throughout the simulation. CID 71423 (green) and CID 5318517 (brown) show moderate fluctuations but maintain an average Rg of ~2.10 nm, suggesting well-preserved structural integrity. In contrast, CID 65664 (orange) shows slightly higher variability and a transient peak above 2.20 nm during the early stages of the simulation, indicating localized unfolding or flexibility in specific regions (Figure 7A).

The Rg profiles of TryR-ligand complexes show slightly larger values compared to GPDH, fluctuating between 2.44–2.56 nm. CID 97214 (red), CID 6529858 (blue), and CID 5318517 (brown) exhibit the most stable compactness, with Rg values tightly clustered around 2.50 nm, reflecting strong protein-ligand interactions and stable structural folding. CID 459963 (purple) shows moderate fluctuations with occasional peaks near 2.54 nm, indicating localized flexibility in certain protein regions. CID 71423 (green) and CID 65664 (orange) demonstrate higher variability, with Rg values occasionally exceeding 2.56 nm, which may reflect transient structural rearrangements or less stable binding (Figure 7B).

The Rg analysis highlights the overall structural stability of GPDH and TryR in the presence of ligands, with compactness preserved across the simulation. CID 97214 consistently demonstrate the most stable protein-ligand interactions for both targets, reflecting their potential as effective inhibitors. CID 65664 exhibits the highest variability, suggesting weaker interactions and potential structural instability. These findings emphasize the structural integrity of key ligands and their suitability for further studies targeting GPDH and TryR.

### 3.6. PCA

**Figure 8:**
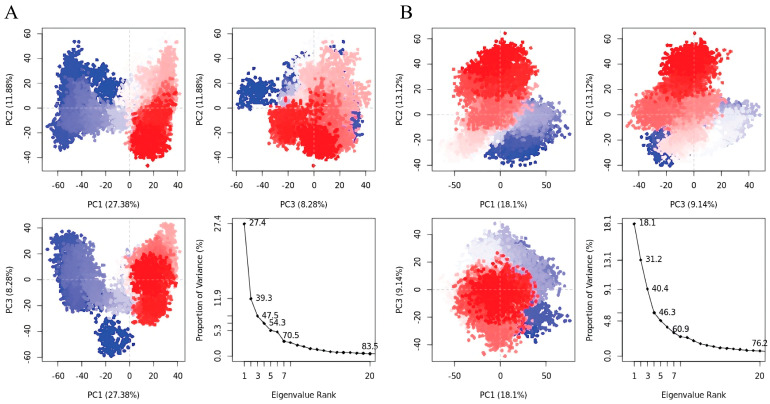
Principal Component Analysis (PCA) results for GPDH and TryR complexes with ligand CID 97214. (**A**) GPDH complexed with ligand CID 97214. PC1 vs. PC2: PC1 explains 27.38% of the variance, and PC2 explains 11.88%, indicating distinct conformational clusters in the protein-ligand dynamics. PC2 vs. PC3: PC2 (11.88%) and PC3 (8.28%) reveal the variance in a 3D conformational space, highlighting the protein’s flexibility. PC1 vs. PC3: Variance between PC1 and PC3 indicates further conformational differences induced by ligand binding. cumulative variance explained by PC1, PC2, and PC3 (27.38%, 11.88%, and 8.28%, respectively) illustrates that these components account for nearly half of the total variability. (**B**) TryR complexed with ligand CID 97214. PC1 vs. PC2: PC1 explains 18.1%, and PC2 explains 13.12%, demonstrating distinct but overlapping conformational states. PC2 vs. PC3: PC2 (13.12%) and PC3 (9.14%) provide insights into conformational dynamics in a 3D space. PC1 vs. PC3: Distribution along PC1 and PC3 showcases the ligand’s subtle impact on TryR structure. Variance explained by PC1, PC2, and PC3 (18.1%, 13.12%, and 9.14%, respectively) suggests these components capture the most significant conformational variability. Colors denote group assignment: blue = Group 1, red = Group 2; lighter/washed-out shades indicate samples with more intermediate/ambiguous positioning between the two groups.

Principal Component Analysis (PCA) was employed to investigate the conformational dynamics of GPDH and TryR proteins complexed with ligand CID 97214 as it showed stability in both of the proteins and have a potent candidate for dual inhibitor. The PCA suggests that ligand binding restricts large-scale breathing motions of the protein, yielding a more compact conformational space and supporting greater complex stability, likely by stabilizing flexible loop regions near the binding pocket. The results revealed significant differences in the stability and flexibility of the protein-ligand complexes:

The PCA of GPDH complexed with 97214 highlighted distinct conformational clusters, with PC1 explaining a substantial 27.38% of the variance (Table 4). This indicates a strong influence of the ligand on the protein’s conformational states, suggesting stable binding. The distribution across PC1, PC2, and PC3 further underscores the protein’s flexibility and adaptability in response to the ligand. The PCA results of TryR complexed with 97214 demonstrated overlapping conformational states, with PC1 explaining 18.1% of the variance. This suggests a more subtle impact of the ligand on the protein structure compared to GPDH. The variability observed in PC2 and PC3 highlights potential shape complementarity and flexibility in the binding interaction. These findings suggest that ligand CID 97214 induces notable conformational changes in both GPDH and TryR, albeit to different extents. The pronounced clustering in GPDH implies a stronger ligand influence, making it a promising target for further structural and dynamic analysis. Conversely, the overlapping states in TryR may reflect inherent flexibility, which could be further explored to understand ligand adaptability within its binding site (Figure 8).

### 3.7. Covariance Analysis

**Figure 9:**
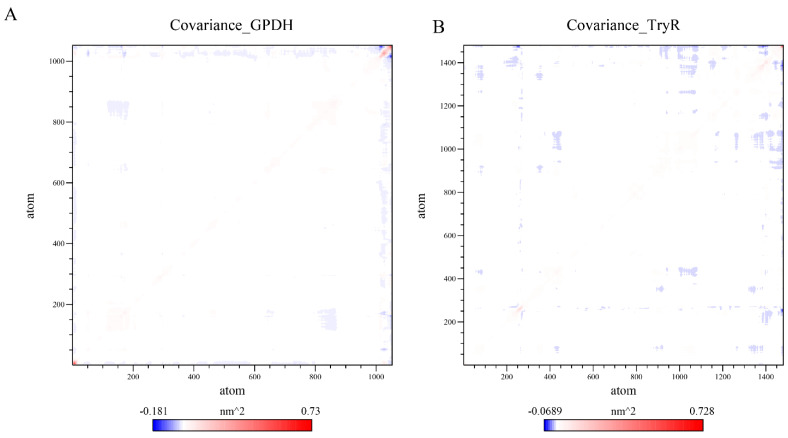
Covariance matrices of GPDH (**A**) and TryR (**B**) backbone atoms complexed with CID 97214 over a 100 ns molecular dynamics simulation.

Covariance analysis provides insights into the dynamic behavior of protein residues by evaluating correlated (positive covariance) and anti-correlated (negative covariance) motions. The covariance maps indicate that stable complexes show more organized correlated motions and reduced disruptive anti-correlations, implying that ligand binding promotes coordinated residue movements that help maintain binding-site geometry. For GPDH (Figure 9A) and TryR (Figure 9B) complexed with CID 97214, the covariance matrices quantify atomic motions, reflecting how ligand binding impacts protein stability and flexibility. The matrix elements range from highly anti-correlated (−0.181 nm^2^ for GPDH, −0.0689 nm^2^ for TryR) to strongly correlated (0.73 nm^2^ for GPDH, 0.728 nm^2^ for TryR), capturing intricate residue-level interactions.

The covariance matrix for GPDH complexed with CID 97214 reveals localized regions of correlated and anti-correlated atomic motions, primarily concentrated near specific backbone regions. The covariance values range from strongly anti-correlated (−0.181 nm^2^) to highly correlated (0.73 nm^2^). Significant correlated motions are observed between atom indices 800–1000, highlighting a rigid region near the binding pocket that remains stabilized by the ligand. This stability is likely to enhance the structural integrity of GPDH during the simulation. Moderate correlated interactions are also observed between indices 200–400, suggesting cooperative motions that maintain overall protein stability. Anti-correlated motions, on the other hand, are weaker and occur mainly between indices 1–200 and 800–1000, reflecting minimal opposing movements in distal regions. The overall motion profile suggests that CID 97214 reinforces the stability of the binding pocket while preserving the flexibility needed for GPDH functionality. The covariance matrix for TryR complexed with CID 97214 shows a broader range of correlated and anti-correlated motions compared to GPDH, indicating greater structural flexibility. The covariance values range from −0.0689 nm^2^ (anti-correlated) to 0.728 nm^2^ (correlated). Strong correlated motions are prominent between atom indices 1000–1400, reflecting stable and cooperative interactions in structural domains associated with ligand binding and enzymatic activity. Additional moderate correlations (~0.3–0.5 nm^2^) are observed between indices 400–600, suggesting stable but adaptable interactions in key regions. Anti-correlated motions are more pronounced in TryR, particularly between 1–200 and 1200–1400, where opposing movements indicate flexibility in distant regions of the protein. These motions highlight TryR’s inherent adaptability, with regions of flexibility complementing the stabilizing influence of CID 97214 on the binding pocket. This dynamic interplay enables TryR to accommodate ligand binding while maintaining its functional motions.

## 4. Discussion

Leishmaniasis ranks as the second most prevalent protozoan disease, marked by its widespread occurrence and high mortality rate. Among the potential drug targets for combating Leishmania [26,27], Glycerol-3-Phosphate Dehydrogenase (GPDH) and Trypanothione Reductase (TryR) have been identified as critical targets, as they play essential roles in the parasite’s metabolic pathways and oxidative stress regulation, making them attractive candidates for therapeutic intervention [28].

Chemotherapy remains the cornerstone for leishmaniasis treatment; however, its effectiveness is increasingly undermined by the growing prevalence of drug resistance. This has become a significant concern in several countries, including India. Resistance arises from diverse mechanisms, such as impaired drug reduction or activation, decreased drug uptake, and enhanced efflux or sequestration of active compounds [29,30,31]. In kinetoplastid parasites like Trypanosoma and Leishmania, the absence of catalase and glutathione (GSH) peroxidase renders them reliant on the tryparedoxin pathway for hydroperoxide removal and oxidative stress regulation. This dependency highlights a potential vulnerability that can be exploited for therapeutic interventions. Developing new drugs targeting such essential pathways could provide breakthroughs in leishmaniasis treatment, freeing the immune system and physiological processes from the constraints imposed by existing therapeutic failures [32,33,34].

The results demonstrate the potential of computational methods to rapidly and effectively identify promising lead compounds. Key findings include the identification of N′-Methoxy-4-[5-[4-[(E)-N′-Methoxycarbamimidoyl]Phenyl]-2-Furyl]Benzamidine (CID: 6529858), Eupatorin (CID: 97214), and Beta-Artesunate (CID: 65664) as potent candidates based on their binding affinities, selectivity, and favorable ADMET profiles. Notably, CID 6529858 exhibited exceptional selectivity for parasitic GPDH, with a significant binding affinity relative to human GPDH, underscoring its potential to minimize off-target effects. The inclusion of human GPDH docking provides an early computational check for off-target liability, which is important given the structural similarity between parasite and host enzymes. The observed ΔDocking values for the lead compounds (approximately −1.0 to −1.7 kcal/mol) suggest a parasite-favored trend (Table 3). If such energy gaps were to translate into true binding free-energy differences, they could correspond to roughly ~5- to ~18-fold differences in affinity; however, because docking scores neglect important effects (e.g., full solvation, receptor flexibility) and are method-dependent, these selectivity estimates should be considered hypothesis-generating. Eupatorin emerged as a dual inhibitor with strong binding stability and minimal fluctuations across both targets during molecular dynamics simulations, highlighting its therapeutic promise. The integration of human GPDH as negative control was pivotal in evaluating the selectivity of the inhibitors, a critical factor in minimizing toxicity. The structural similarity between human and *Leishmania* GPDH underscores the importance of selective inhibitor design, a challenge addressed effectively through this study’s computational pipeline. The observed binding differences between human and parasitic enzymes validate the specificity of the selected compounds and highlight their clinical relevance. Despite these promising results, the study acknowledges the limitations of in silico approaches, including the lack of experimental validation. Future Studies to validate the computational hits, the top compounds should be tested in enzyme inhibition assays against *Leishmania* GPDH (IC_50_/Ki), alongside a counter-assay with human GPDH to assess selectivity. Active candidates can then be confirmed using binding assays (e.g., DSF, SPR, or MST) and evaluated in cell-based anti-leishmanial models (promastigote/amastigote) with host–cell cytotoxicity testing to estimate a therapeutic window, followed by basic ADME profiling and in vivo efficacy studies for the most promising leads. 

Recent studies have explored the inhibition of Trypanothione Reductase (TR) as a therapeutic strategy against the Leishmania species. For instance, Matadamas-Martínez et al. (2019) [35] conducted computational and biological studies to identify inhibitors of Leishmania mexicana TR. They performed molecular docking of 20 compounds from the ZINC database and selected five for further evaluation. These compounds inhibited recombinant TR activity by 32.9% to 40.1% and demonstrated leishmanicidal activity against *L. mexicana* promastigotes. Notably, compound ZINC12151998 exhibited the highest leishmanicidal activity with an IC_50_ of 58 µM, outperforming the standard drug glucantime. This compound provides a promising starting point for developing new anti-Leishmania drugs [35]. Additionally, Raj et al. (2020) provided an overview of biochemically characterized drug targets in the metabolic pathways of Leishmania, emphasizing the importance of enzymes like TryR and GPDH in the parasite’s survival and pathogenicity [36]. Another review has mentioned the use of gold complexes as TR inhibitors. They found that these complexes were potent inhibitors of TR and exhibited significant activity against *Leishmania infantum* and *Leishmania braziliensis* intracellular amastigotes. The treatment led to mitochondrial damage and oxidative stress in the parasites, indicating the potential of these gold complexes as oral drug candidates for leishmaniasis treatment [37].

This study provides a comprehensive framework for the identification of selective dual inhibitors targeting *Leishmania* TryR and GPDH. By addressing critical limitations in existing therapies, including drug resistance and toxicity, these findings contribute to the development of novel, effective, and safe therapeutics for leishmaniasis. The integration of computational tools, combined with rigorous validation, offers a promising avenue for accelerating drug discovery in neglected tropical diseases. A key limitation is that in silico ADMET tools provide probabilistic estimates and cannot replace experimental toxicology; thus, in vitro cytotoxicity and organ-specific safety assays remain necessary to validate the safety profile of shortlisted compounds. While docking is useful for rapid screening, predicted scores are sensitive to scoring functions and simplified representations of solvation and receptor flexibility; thus, the docking results in this study are best interpreted as preliminary prioritization that requires experimental confirmation. In conclusion, Leishmaniasis remains a major public health burden in endemic regions, where access to safe, affordable, and effective treatments is often limited. Therefore, identifying drug-like inhibitors of *Leishmania* GPDH may support future development of more accessible therapies, while the present findings should be considered hypothesis-generating until experimentally validated.

## 5. Conclusions

This study highlights TryR and GPDH as promising dual targets for disrupting essential redox balance and energy metabolism in *Leishmania*, supporting a multi-target strategy that may help reduce resistance risk. The prioritized compounds, particularly Eupatorin, represent plausible starting points for developing safer and more effective anti-leishmanial leads with lower off-target liability. Importantly, these findings are most relevant for endemic regions where treatment options remain limited and the need for affordable, accessible therapies is urgent. Future work should validate these candidates experimentally and refine them through optimization to advance toward translational applicability for leishmaniasis control. 

## Figures and Tables

**Table 1:** Parameters for AMDET analysis.

Parameter	Description	Threshold/Criteria
GI Absorption	Predicted gastrointestinal absorption efficiency.	High
BBB Permeability	Ability of the compound to penetrate the blood–brain barrier.	Should be NO an
Lipinski’s Rule of Five	Rule assessing drug-likeness for oral bioavailability.	≤5 H-bond donors, ≤10 H-bond acceptors, MW ≤ 500 Da, LogP ≤ 5.
XLogP	Logarithmic partition coefficient for lipophilicity.	≤5 (preferable range).
Rotatable Bonds	Number of rotatable bonds, indicating molecular flexibility.	≤10 (preferred for oral bioavailability).
H-Bond Donors	Number of hydrogen bond donors.	≤5 (preferred for oral drugs).
H-Bond Acceptors	Number of hydrogen bond acceptors.	≤10 (preferred for oral drugs).
Hepatotoxicity	Potential for liver toxicity as predicted by pkCSM.	Preferably Non-Toxic.
Ames Test (Mutagenicity)	Likelihood of causing genetic mutations.	Preferably non-mutagenic.
hERG I Inhibitor	Prediction of inhibition of hERG potassium channels (hERG I).	Preferably non-inhibitor.
hERG II Inhibitor	Prediction of inhibition of hERG potassium channels (hERG II).	Preferably non-inhibitor.
Skin Sensitivity	Predicted potential to cause skin sensitization reactions.	Preferably non-sensitizer.

**Table 2:** ADMET analysis of final sorted ligand.

Chemical	CID	GI Absorption	Lipinski Rules Violation (Out of 5)	BBB Permeant	AMES Toxicity	Hepatotoxicity	Skin Sensitisation
Pentamidine	4735	High	0	No	No	No	No
Arsthinol	8414	High	0	No	No	No	No
Haloxon	9454	High	0	No	No	No	No
Kainic acid	10255	High	0	No	No	No	No
8-Hydroxy-7-iodo-5-quinolinesulfonic acid	11043	High	0	No	No	No	No
Propamidine	64949	High	0	No	No	No	No
Beta-Artesunate	65664	High	0	No	No	No	No
Tiazuril	71423	High	0	No	No	No	No
Tioxidazole	72157	High	0	No	No	No	No
Panamidin dihydrochloride	80467	High	0	No	No	No	No
Fosthiazate	91758	High	0	No	No	No	No
EUPATORIN	97214	High	0	No	No	No	No
4-Oxo-4-[(1,5,9-trimethyl-11,14,15,16-tetraoxatetracyclo[10.3.1.04,13.08,13]hexadecan-10-yl)oxy]butanoic acid	105031	High	0	No	No	No	No
4-Oxo-4-[[(1S,4S,5R,8S,9R,10R,12R,13R)-1,5,9-trimethyl-11,14,15,16-tetraoxatetracyclo[10.3.1.04,13.08,13]hexadecan-10-yl]oxy]butanoic acid	156252	High	0	No	No	No	No
1H-Indole-4,6-dicarboximidamide, 2-phenyl-	156646	High	0	No	No	No	No
CIRSILINEOL	162464	High	0	No	No	No	No
Levamisole Phosphate	198119	High	0	No	No	No	No
Spirotriazine	210321	High	0	No	No	No	No
Tosulur	216264	High	0	No	No	No	No
Anisomycin	253602	High	0	No	No	No	No
2,5-Bis(4-amidinophenyl)furan-bis-O-methylamidoxime; 4,4′-(2,5-Furandiyl)bis[N-methoxybenzenecarboximidamide]; 4,4′-(2,5-Furandiyl)bis[N-methoxybenzenecarboximidamide	459963	High	0	No	No	No	No
Thenium Closylate	498092	High	0	No	No	No	No
Spirotriazine Hydrochloride	3057054	High	0	No	No	No	No
Stibosamine	3081396	High	0	No	No	No	No
Chrysoplenol D	5280699	High	0	No	No	No	No
Chrysosplenetin	5281608	High	0	No	No	No	No
Casticin	5315263	High	0	No	No	No	No
Andrographolide	5318517	High	0	No	No	No	No
Artemetin	5320351	High	0	No	No	No	No
Succinyl dihydroartemisinin	5464098	High	0	No	No	No	No
Pafuramidine	5480200	High	0	No	No	No	No
N′-methoxy-4-[5-[4-[(E)-N′-methoxycarbamimidoyl]phenyl]-2-furyl]benzamidine	6529858	High	0	No	No	No	No
Artesunate	6917864	High	0	No	No	No	No
4-oxo-4-{[(5as,6R,8as,9R,10S,12R,12ar)-3,6,9-trimethyldecahydro-3,12-epoxy[1,2]dioxepino[4,3-i]isochromen-10-yl]oxy}butanoic acid	16394563	High	0	No	No	No	No
Sodium artesunate	44410736	High	0	No	No	No	No
4-oxo-4-[[(1S,4S,8S,9R)-1,5,9-trimethyl-11,14,15,16-tetraoxatetracyclo[10.3.1.04,13.08,13]hexadecan-10-yl]oxy]butanoic acid	49769200	High	0	No	No	No	No
4-oxo-4-[[(4S,5R,8S,9R,12R,13R)-1,5,9-trimethyl-11,14,15,16-tetraoxatetracyclo[10.3.1.04,13.08,13]hexadecan-10-yl]oxy]butanoic acid	53486426	High	0	No	No	No	No
Dihydroartemisinin alpha-hemisuccinate sodium salt	71300409	High	0	No	No	No	No
4-oxo-4-[[(1R,5R,9R,10S,13R)-1,5,9-trimethyl-11,14,15,16-tetraoxatetracyclo[10.3.1.04,13.08,13]hexadecan-10-yl]oxy]butanoic acid	91746179	High	0	No	No	No	No
4-oxo-4-[[(1R,4S,5S,8S,9S,10R,12S,13R)-1,5,9-trimethyl-11,14,15,16-tetraoxatetracyclo[10.3.1.04,13.08,13]hexadecan-10-yl]oxy]butanoic acid	129009911	High	0	No	No	No	No
4-oxo-4-[[(13R)-1,5,9-trimethyl-11,14,15,16-tetraoxatetracyclo[10.3.1.04,13.08,13]hexadecan-10-yl]oxy]butanoic acid	146160078	High	0	No	No	No	No

**Table 3:** Binding Affinities and Selectivity of Ligands Against Leishmania Trypanothione Reductase (TryR) and Glycerol-3-Phosphate Dehydrogenase (GPDH).

Chemical	Ligand	TryR (kcal/mol)	GPDH Leishmania (kcal/mol)	GPDH Human (kcal/mol)	Affinity Difference (Gpdh_Leishmania and Gpdh_Human)
Artesunate	6917864	−8.2	−7.6	−7.5	−0.1
4-Oxo-4-[[(1s,4s,8s,9r)-1,5,9-Trimethyl-11,14,15,16-Tetraoxatetracyclo[10.3.1.04,13.08,13]Hexadecan-10-Yl]Oxy]Butanoic Acid	49769200	−8.2	−8	−7.8	−0.2
2,5-Bis(4-Amidinophenyl)Furan-Bis-O-Methylamidoxime; 4,4′-(2,5-Furandiyl)Bis[N-Methoxybenzenecarboximidamide]; 4,4′-(2,5-Furandiyl)Bis[N-Methoxybenzenecarboximidamide	459963	−8.1	−8.5	−7.5	−1
4-Oxo-4-[[(4s,5r,8s,9r,12r,13r)-1,5,9-Trimethyl-11,14,15,16-Tetraoxatetracyclo[10.3.1.04,13.08,13]Hexadecan-10-Yl]Oxy]Butanoic Acid	53486426	−8.1	−8.2	−7.5	−0.7
4-Oxo-4-[[(1r,5r,9r,10s,13r)-1,5,9-Trimethyl-11,14,15,16-Tetraoxatetracyclo[10.3.1.04,13.08,13]Hexadecan-10-Yl]Oxy]Butanoic Acid	91746179	−8.1	−8.2	−7.5	−0.7
Andrographolide	5318517	−8	−8.4	−7.4	−1
4-Oxo-4-{[(5as,6R,8as,9R,10S,12R,12ar)-3,6,9-Trimethyldecahydro-3,12-Epoxy[1,2]Dioxepino[4,3-I]Isochromen-10-Yl]Oxy}Butanoic Acid	16394563	−8	−7.9	−7.5	−0.4
Succinyl Dihydroartemisinin	5464098	−7.9	−8	−7.9	−0.1
Tiazuril	71423	−7.8	−8.7	−7.5	−1.2
Anisomycin	253602	−7.7	−7.4	−7.8	0.4
Beta-Artesunate	65664	−7.5	−8.2	−7.1	−1.1
Eupatorin	97214	−7.5	−8.2	−7.2	−1
N′-Methoxy-4-[5-[4-[(E)-N′-Methoxycarbamimidoyl]Phenyl]-2-Furyl]Benzamidine	6529858	−7.5	−8.9	−7.2	−1.7
4-Oxo-4-[[(1r,4s,5s,8s,9s,10r,12s,13r)-1,5,9-Trimethyl-11,14,15,16-Tetraoxatetracyclo[10.3.1.04,13.08,13]Hexadecan-10-Yl]Oxy]Butanoic Acid	129009911	−7.5	−8.4	−7.7	−0.7
Cirsilineol	162464	−7.4	−8	−7.7	−0.3
Spirotriazine	210321	−7.4	−7.6	−7.1	−0.5
Chrysoplenol D	5280699	−7.3	−7.4	−7.3	−0.1
Chrysosplenetin	5281608	−7.3	−7.2	−7.3	0.1
Casticin	5315263	−7.3	−7	−6.8	−0.2
Pentamidine	4735	−7.2	−7.5	−6.1	−1.4
1h-Indole-4,6-Dicarboximidamide, 2-Phenyl-	156646	−7.2	−7.6	−8.4	0.8
Artemetin	5320351	−7.1	−7	−6.7	−0.3
Pafuramidine	5480200	−6.7	−8.5	−7.8	−0.7
Tioxidazole	72157	−6.3	−6.4	−6.1	−0.3
Propamidine	64949	−6.2	−7	−6.1	−0.9
Tosulur	216264	−6.2	−6.3	−7.5	1.2
Haloxon	9454	−5.9	−6.6	−6.6	0
8-Hydroxy-7-Iodo-5-Quinolinesulfonic Acid	11043	−5.8	−5.8	−6.2	0.4
Kainic Acid	10255	−5.4	−6.1	−6.6	0.5
Fosthiazate	91758	−5	−5.1	−4.5	−0.6

**Table 4:** PCA Components for Protein-Ligand Complexes.

Complex	PC1 Explained (%)	PC2 Explained (%)	PC3 Explained (%)	Total Variance	PC1 vs. PC2 (Distribution)	PC2 vs. PC3 (Distribution)	PC1 vs. PC3 (Distribution)
GPDH_CID97214	27.38%	11.88%	8.28%	47.54%	Distinct conformational clusters	3D view, variance by PC2 and PC3	Distribution along PC1 and PC3
TryR_CID97214	18.1%	13.12%	9.14%	40.36%	Overlapping conformational states	Variance by PC2 and PC3	Subtle variations along PC1 & PC3

## Data Availability

The original contributions presented in this study are included in the article/Supplementary Materials. Further inquiries can be directed to the corresponding author.

## Supplementary Materials

The following supporting information can be downloaded at: https://www.mdpi.com/article/10.3390/microorganisms14020407/s1, File S1: ADMET analysis.
